# Changes in the Clinical Practice of Mental Health Service Providers Throughout the COVID-19 Pandemic: Longitudinal Questionnaire Study

**DOI:** 10.2196/50303

**Published:** 2024-04-29

**Authors:** Milena Gotra, Katharine Lindberg, Nicholas Jasinski, David Scarisbrick, Shannon Reilly, Jonathan Perle, Liv Miller, James Mahoney III

**Affiliations:** 1 Department of Behavioral Medicine and Psychiatry Rockefeller Neuroscience Institute West Virginia University School of Medicine Morgantown, WV United States; 2 Atrium Health Charlotte, NC United States; 3 Department of Neuroscience Rockefeller Neuroscience Institute West Virginia University School of Medicine Morgantown, WV United States; 4 Department of Neurology University of Virginia Health Charlottesville, VA United States

**Keywords:** COVID-19 pandemic, mental health, social worker, psychologist, neuropsychologist, academic medical center, community mental health, private practice, Veteran’s Affairs hospital, longitudinal questionnaire study, COVID-19, implementation, telemental health, hybrid model, availability

## Abstract

**Background:**

The COVID-19 pandemic impacted the practices of most mental health providers and resulted in a rapid transition to providing telemental health services, changes that were likely related to stay-at-home policies as well as increased need for services.

**Objective:**

The aim of this study was to examine whether these changes to practice have been sustained over time throughout the course of the COVID-19 pandemic and whether there are differences among mental health provider type and setting. We hypothesized that there would be an increase in the number of patients seen in person after the initial surge of the pandemic in spring 2020 and subsequent discontinuation of stay-at-home policies, though with continued implementation of telemental health services across settings.

**Methods:**

This study surveyed 235 of the 903 mental health providers who responded to a survey in spring 2020 (Time point 1) and at a 1-year follow-up in spring 2021 (Time point 2). Differences in practice adjustments, factors related to telemental health, and number of patients seen were examined across provider type (social worker, psychologist, neuropsychologist) and setting (academic medical center [AMC], community mental health, private practice, and Veterans Affairs hospital).

**Results:**

From Time point 1 to Time point 2, there was a small but significant increase in the overall number of providers who were implementing telehealth (191/235, 81% to 204/235, 87%, *P*=.01) and there was a significant decline in canceled or rescheduled appointments (25%-50% in 2020 to 3%-7% in 2021, *P*<.001). Psychologists and providers working at AMCs reported decreased difficulty with telehealth implementation (*P*<.001), and providers working at AMCs and in private practice settings indicated they were more likely to continue telehealth services beyond spring 2021 (*P*<.001). The percent of time working remotely decreased overall (78% to 59%, *P*<.001), which was most notable among neuropsychologists and providers working at an AMC. There was an overall increase in the average number of patients seen in person per week compared with earlier in the pandemic (mean 4.3 to 8.7, *P*<.001), with no change in the number of patients seen via telehealth (mean 9.7 to 9.9, *P*=.66).

**Conclusions:**

These results show that the rapid transition to telemental health at the onset of the COVID-19 pandemic in spring 2020 was sustained over the next year, despite an overall increase in the number of patients seen in person. Although more providers reported returning to working on-site, over 50% of providers continued to use a hybrid model, and many providers reported they would be more likely to continue telemental health beyond spring 2021. This suggests the continued importance and reliance on telemental health services beyond the acute pandemic phase and has implications for future policies regulating the availability of telemental health services to patients.

## Introduction

In March 2020, the World Health Organization declared the COVID-19 outbreak, caused by SARS-CoV-2 infection, a global pandemic [1]. Governments around the globe began implementing various strategies to contain the spread of COVID-19 including recommendations for social distancing, restrictions on in-person gatherings and contact, and international travel limitations, among many others [2,3]. In particular, the recommendations for limited interpersonal contact presented mental health service providers with a unique challenge in an attempt to maintain adequate access to services during a time in which mental health care needs were on the rise [4-7].

As a result of these challenges, numerous researchers began exploring the impact of the COVID-19 pandemic on the provision of mental health services [8,9]. Duden et al [9] conducted a systematic review of 29 international studies documenting the COVID-19 pandemic’s impact on mental health services from December 2019 through March 2022. They indicated 9 major topic areas in mental health services impacted by the COVID-19 pandemic, including (1) lack of preparedness versus timely response and flexible solutions; (2) changes in access, referral, and admission; (3) impacts on outpatient, community, and psychosocial services; (4) inpatient reorganization of hospital psychiatric units and acute wards; (5) diagnostic and therapeutic adaptations (including rapid deployment of telemental health services); (6) effects on medication; (7) infection control measures; (8) changes in patients’ demands, engagement, and mental health; and (9) impacts on staff and team.

Although there have been longitudinal assessments of the impact of the pandemic on patients [10-16], there has been a paucity of published longitudinal data to examine how provider practices have changed throughout the course of the pandemic. Although various studies have collected data at different time points during the pandemic, these cross-sectional evaluations offer little in the way of understanding how providers’ perceptions and activities were modified over time, a limitation which has previously been recognized [8,17-22]. Given the ebb and flow of different COVID-19 variants and changing guidelines in dealing with the ongoing pandemic (eg, reduced distancing requirements, lifting of travel restrictions), it is possible, and likely probable, that provider practice adjustments may subsequently change over time.

This manuscript represents a follow-up to the study conducted by Reilly et al [23] in 2020. Briefly, the results from the original investigation found that over 97% of a sample of US mental health providers (n=903) made practice adjustments as a result of the developing pandemic. The most consistent change identified was the increasing use of telemental health appointments, with nearly 80% of respondents adopting this service delivery method. Other findings of note included providers’ generally positive perceptions of telemental health preparedness, the desire of respondents to continue providing telemental health services in the future, reduced rate of patient contacts, and the variations in practice modifications made by different types of US mental health professionals. The broader mental health practice literature has suggested that US mental health providers across disciplines are moving to a hybrid model of mixed telemental health and in-person services [8,9,24-26].

This study sought to address the impact of the continued COVID-19 pandemic by examining longitudinal data related to changes in the clinical practice of US mental health providers from December 2019 through March 2021. We predicted that, given the progressive relaxation of stay-at-home policies and social restrictions, there would be an overall increase in the number of patients seen and fewer cancellations of appointments from spring 2020 to spring 2021. We further predicted there would be continued high adoption of telemental health services and that providers would have easier access to telemental health infrastructure (eg, IT services, technology). Among provider type, we predicted that neuropsychologists would see fewer patients virtually and would have a greater increase in in-person services, likely related to greater difficulty performing services such as neuropsychological testing via telehealth.

## Methods

### Recruitment

In March 2021, participants who completed the initial survey [23] and indicated at that time that they were interested in participating in possible follow-up surveys (and provided their email address) were contacted via email. The recruitment email included a Qualtrics survey link. All questions were optional, and participants were informed they could discontinue participation at any time. Data collection was completed, and the survey was closed in April 2021.

### Data Collection

The follow-up survey was similar in structure and content to the initial survey [23]. Participants were asked to provide information about their demographics, patient populations, practice adjustments in response to COVID-19, perceptions of their employer’s response, and their emotional response and perceptions regarding the COVID-19 pandemic (see Multimedia Appendix 1). Additionally, participants were asked about changes in their status since the initial study (eg, household income, employment, training) as well as questions regarding coping strategies and vaccination status. For the Time point 2 (T2) study, precautionary measures were not included as a separate practice adjustment.

For applicable questions, participants were asked about their practices in December 2020 (to match the initial survey that asked about practice in December 2019) and their “current” practices (ie, when they completed the survey between March 2021 and April 2021). Of the 626 initial survey participants who provided their email for follow-up work and were contacted regarding the follow-up survey, 235 participants were included in the final sample. Data were excluded if participants completed less than 66% of the follow-up survey (n=37). We excluded 3 participants because they did not have complete survey data for both time points.

### Data Preparation

Less than 5% of the data were missing for each variable of interest, with the exception of the number of patients seen via telehealth in December 2019 (21/235, 8.9%) and percent of time working remotely during the initial survey (18/235, 7.7%) and this follow-up survey (34/235, 14.5%). Missing data were addressed using pairwise deletion. Similar to the initial study, 3 participants who identified as marriage and family therapists were recoded as a master’s-level provider given the small sample size for this category. Participants with a discrepancy between their provider type and highest education (eg, provider with a master’s degree who identified as a psychologist or doctoral-level provider, n=6) were recoded to reflect their level of education. Square root transformations were conducted on continuous variables to address normality and reduce outliers.

### Statistical Analysis

Analyses were conducted in SPSS Version 26 (IBM Corp). Outcome variables were compared between the initial survey (Time point 1 [T1]) and follow-up survey (T2) for the overall sample and across provider type (social worker/master’s-level provider vs psychologist/doctoral-level provider vs neuropsychologist) and setting (academic medical center [AMC] vs community mental health vs private practice [PP] vs Veterans Affairs hospital). We chose to limit our analyses to these settings and provider types as these were the largest sample sizes that were also examined in the T1 study [23]. The related-sample McNemar test was used to compare binomial variables among groups (eg, practice adjustments, whether participants were implementing telehealth, whether participants had easy access to IT services). The Wilcoxon signed rank test was used to compare differences in variables ranked on a Likert scale (eg, difficulty of implementing telehealth appointments, likelihood of providing telehealth services in the future). Paired sample *t* tests were used to compare changes from T1 to T2 for continuous variables for the overall sample. Differences across provider type and setting from T1 to T2 were evaluated using a mixed ANOVA (time x group) for continuous variables, with Bonferroni-corrected post hoc tests. Continuous variables included the percent of time working remotely and the number of in-person, remote, and total patients seen weekly. The number of patients seen was examined across 4 time points, including December 2019 (T1), March 2020 to April 2020 (T1), December 2020 (T2), and March 2021 to April 2021 (T2). Percent of time working remotely was calculated only for those who reported a value greater than 0% (n=135). Analyses were evaluated as significant with a false discovery rate correction for multiple comparisons [27].

### Ethical Considerations

This study was determined to be exempt following the research ethics review by the Institutional Review Board of West Virginia University. Participants consented to participate by submitting their survey responses. Participant data were deidentified and matched with the unique identifier from the first survey. Participants were not compensated for completing the survey.

## Results

### Demographic Characteristics

Participant demographic characteristics are presented in Tables 1 and  2. There were no significant differences in demographic characteristics of participants who responded to the follow-up study compared with the full sample for the initial study [23]. There was a slightly higher proportion of psychologists who responded to the follow-up study (111/235, 47.2% compared with 367/903, 40.64%), though other professional characteristics were comparable. At the time of the T2 study, 15% (34/235) of respondents reported a change of work status since April 2020, due to change of jobs, fewer overall hours, starting new training (ie, new practicum placement, starting internship and postdoctoral fellowship), starting a new position, being laid off, and changing practice settings. Of the respondents, 17% (41/235) reported a change to their provider type since April 2020, and 9% (22/235) of respondents reported moving or changing the state they are employed in since April 2020.

**Table 1:** Demographic characteristics of participants who completed the follow-up survey (n=235).

Characteristics		Results
Age (years), mean (SD)		38.7 (10.4)
**Sex/gender, n (%)**		
	Woman	205 (87.2)
	Man	30 (12.8)
**Race/ethnicity, n (%)**		
	White	212 (90.2)
	Black/African American	7 (3.0)
	Asian/Asian American	6 (2.6)
	Hispanic/Latinx	6 (2.6)
	Multiracial	4 (1.7)
**Sexual orientation, n (%)**		
	Heterosexual	199 (84.7)
	Bisexual	17 (7.2)
	Lesbian/gay	8 (3.4)
	Other (ie, queer, fluid, pansexual)	11 (4.7)
**Region, n (%)**		
	South	106 (45.1)
	Midwest	56 (23.8)
	West	43 (18.3)
	Northeast	30 (12.8)
**Work status, n (%)**		
	Full-time	179 (76.2)
	Part-time	20 (8.5)
	Trainee	35 (14.9)
	Not currently employed	1 (0.4)

**Table 2:** Professional characteristics of respondents who completed the follow-up survey (n=235).

Characteristics		Results, n (%)
**Provider type**		
	Psychologist, doctoral-level therapist, counselor	111 (47.2)
	Neuropsychologist	42 (17.9)
	Trainee (ie, practicum student, predoctoral intern, postdoctoral fellow)	35 (14.9)
	Social worker, master’s-level therapist, counselor	32 (13.6)
	Other (eg, support staff, bachelor-level therapist, physician)	15 (6.4)
**Provider level**		
	Licensed practitioner	146 (62.1)
	Licensed practitioner and board certified in specialty area	38 (16.2)
	Unlicensed practitioner	16 (6.8)
**Current practice setting^a^**		
	Private practice	55 (23.4)
	Academic medical center	52 (22.1)
	Multiple practice settings	34 (14.5)
	Veterans Affairs hospital or military hospital/clinic	25 (10.6)
	Community mental health setting	18 (7.7)
	Psychiatric hospital or facility	10 (4.3)
	General hospital	8 (3.4)
	Rehabilitation hospital or setting	8 (3.4)
	Other (eg, counseling center, training clinic, prison, school, primary care, specialty clinic, outpatient program, nonprofit organization, online program, insurance company)	24 (10.2)
**Age specialty^b^**		
	Adults only (ie, 18 years and older)	122 (51.9)
	Lifespan (ie, pediatrics and adults)	92 (39.1)
	Pediatric only (ie, younger than 18 years)	21 (8.9)

^a^1 respondent did not provide information about their practice setting.

^b^Time 2 results (follow-up survey) were based on those the participants reported at the Time 1 study (initial survey).

### Overall Sample

Characteristics of changes in practice across the total sample are presented in Table 3. Slightly more participants reported implementing telehealth services overall, though the percent of participants who endorsed scheduling virtual instead of in-person visits as a practice adjustment decreased by approximately 10% (169/235) from T1 to T2. Additionally, the percent of time working remotely decreased by approximately 20%. Significantly fewer participants were rescheduling and canceling appointments, though participants endorsed continued use of appointment restrictions (eg, by patient age, medical comorbidity, or recent travel). More participants endorsed no change in practice and reported using a greater variety of “other” practice adjustments, including increasing precautions, adding more patients to their schedule, offering reduced fees, using hybrid virtual and in-person models, and adjusting their test batteries. Although there was no change in ease of access to IT services, participants reported a decrease in difficulty with telehealth implementation from a median of 3 (not easy or difficult) at T1 to 2 (somewhat easy) at T2. Participants reported an increase in the likelihood of continuing telemental health services in the future from a median of 4 (somewhat likely) at T1 to 5 (very likely) at T2.

**Figure 1:**
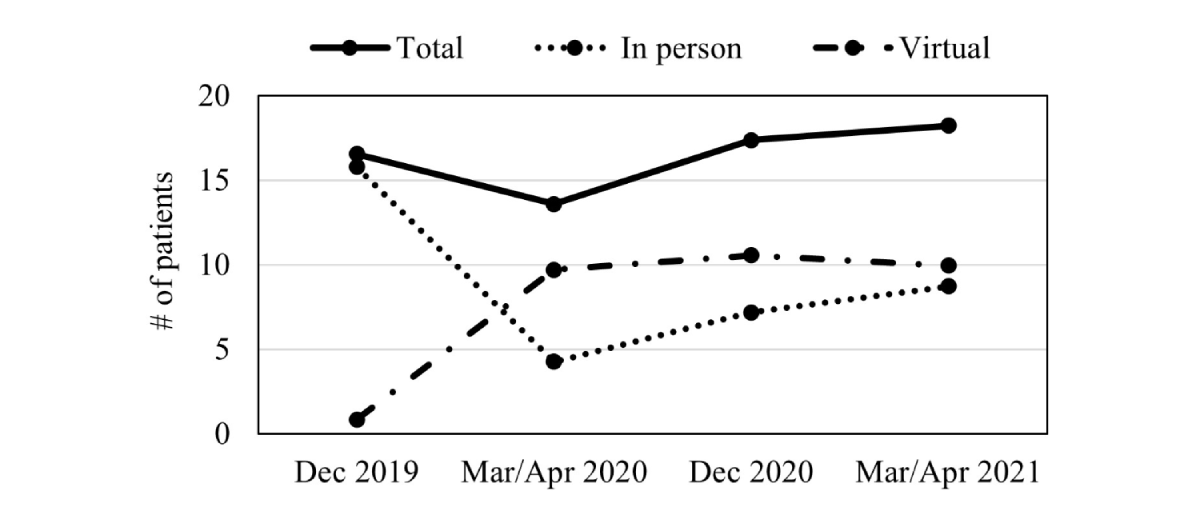
Changes in the number of patients seen weekly for the overall sample.

The total number of patients seen weekly significantly decreased from December 2019 to March 2020/April 2020 (*F*_2.01,463.37_=20.94, *P*<.001; partial η^2^=0.08; mean difference [M_diff_]=3.03, *P*<.001; Figure 1) though returned to pre-COVID-19 levels by December 2020 (M_diff_=–3.66, *P*<.001) and continued to increase by March 2021/April 2021 (M_diff_=–0.91, *P*=.02). The number of patients seen in person showed a similar pattern of initial decline from December 2019 to March 2020/April 2020 (*F*_2.25,481.36_=113.56, *P*<.001; partial η^2^=0.35; M_diff_=11.54, *P*<.001). Although the number of in-person patients subsequently increased (March 2020/April 2020 to December 2020: M_diff_=–3.24, *P*<.001; December 2020 to March 2021/April 2021: M_diff_=–1.36, *P*<.001), they did not return to pre-COVID-19 levels. The number of patients seen virtually initially increased from December 2019 to March 2020/April 2020 (*F*_2.31,476.13_=113.62, *P*<.001; partial η^2^=0.36; M_diff_=–9.04, *P*<.001) and remained stable through March 2021/April 2021.

**Table 3:** Descriptive statistics of changes in clinical practice from Time 1 to Time 2.

Clinical practice			Time 1 study		Time 2 study		Statistic (*df*)		*P* value
**Practice adjustments, n (%)**									
	Telemental health or virtual appointments (vs in-person)	194 (82.6)		169 (71.9)		10.9 (1)^b^		<.001	
	Rescheduling or postponing appointments	118 (50.2)		16 (6.8)		96.2 (1)^b^		<.001	
	Canceling appointments	60 (25.5)		7 (3.0)		47.4 (1)^b^		<.001	
	Restrictions on appointments	32 (13.6)		35 (14.9)		0.08 (1)^b^		.78	
	Other adjustment	6 (2.6)		52 (22.6)		37.5 (1)^b^		<.001	
	N/A^a^ (no change in practice)	6 (2.6)		29 (12.3)		19.4 (1)^b^		<.001	
**Telemental health**									
	Reported currently not implementing telemental health, n (%)	44 (19.3)		31 (13.2)		7.5 (1)^c^		.01	
	Amount of the week working remotely (%), mean (SD)	77.92 (33.6)		58.46 (37.2)		5.1 (134)^c^		<.001	
	Reported easy access to IT services, n (%)	180 (76.6)		177 (75.3)		0.10 (1)^c^		.75	
	Difficulty with telemental health implementation^d^, mean (SD)	3.1 (1.2)		2.7 (1.1)		–5.0^e^		<.001	
	Likelihood of continuing telemental health services^f^, mean (SD)	3.6 (1.4)		4.2 (1.1)		–5.4^e^		<.001	

^a^N/A: not applicable.

^b^Chi-squared.

^c^*t* test.

^d^5-point Likert scale (1=easy or not at all difficult to 5=very difficult).

^e^*Z* score.

^f^5-point Likert scale (1=very unlikely to 5=very likely).

### Provider Type

Characteristics of changes in practice by provider type are presented in Table 4. Only psychologist/doctoral-level providers worked significantly less time per week remotely and had a significant decrease in the percent of participants who reported implementing virtual instead of in-person visits as a practice adjustment. All providers had a decrease in the percent of appointments being rescheduled or postponed, and psychologist/doctoral-level providers and neuropsychologists had a decrease in the percent of appointments being canceled. Psychologist/doctoral-level providers and neuropsychologists reported implementing an increase in “other” practice adjustments. “Other” practice adjustments for psychologist/doctoral-level providers included safety precautions, adding extra appointments, smaller therapy groups, hybrid models, and reducing caseloads. Adjustments for neuropsychologists included safety precautions, limits to the number of family members who can attend appointments, adjustments to test batteries, and hybrid models. Only psychologist/doctoral-level providers had a significant increase in the percent of providers who were not implementing any changes to their current practice. Only psychologist/doctoral-level providers had a significant decrease in difficulty with telemental health implementation and indicated an increased likelihood of continuing telemental health services in the future.

**Table 4:** Descriptive statistics of the changes in clinical practice from Time 1 to Time 2 across provider type.

Clinical practice		Social workers, master’s degree therapists (n=32)			Psychologists, doctoral therapists (n=111)			Neuropsychologists (n=42)		
		Time 1	Time 2	*P* value	Time 1	Time 2	*P* value	Time 1	Time 2	*P* value
**Practice adjustments, n (%)**										
	Telemental health or virtual appointments (vs in person)	29 (90.6)	26 (81.3)	.25	98 (88.3)	84 (75.7)	.002	29 (69.0)	26 (61.9)	.63
	Rescheduling or postponing appointments	13 (40.6)	0	<.001	42 (37.8)	5 (4.5)	<.001	36 (85.7)	8 (19.0)	<.001
	Canceling appointments	6 (18.8)	0	.04^a^	20 (18.0)	4 (3.6)	<.001	14 (33.3)	2 (4.8)	.003
	Restrictions on appointments	3 (9.4)	2 (6.3)	≥.99	12 (10.8)	12 (10.8)	≥.99	11 (26.2)	10 (23.8)	≥.99
	Other adjustment	0	5 (15.6)	.06	1 (2.7)	21 (19.4)	<.001	0	18 (43.9)	<.001
	N/A^b^ (no change in practice)	0	3 (9.4)	.25	2 (1.8)	12 (10.8)	.002	2 (4.8)	6 (14.3)	.13
**Telemental health**										
	Reported not implementing telemental health currently, n (%)	1 (3.3)	2 (6.3)	≥.99	10 (9.2)	9 (8.1)	.69	15 (36.6)	9 (21.4)	.15
	Percent of week working remotely, mean (SD)	81.4 (34.8)	57.7 (40.1)	.04^a^	83.1 (29.8)	62.9 (38.3)	<.001	56.9 (38.5)	43.3 (32.1)	.08
	Easy access to IT services, n (%)	25 (78.1)	24 (75.0)	≥.99	80 (72.1)	80 (72.1)	≥.99	33 (78.6)	35 (83.3)	.69
	Difficulty with telemental health implementation^c^, mean (SD)	2.9 (1.1)	2.5 (1.1)	.04^a^	3.0 (1.2)	2.5 (1.0)	<.001	3.4 (1.3)	3.0 (1.1)	.46
	Likelihood of continuing to provide telemental health services^d^, mean (SD)	3.8 (1.3)	4.12 (1.1)	.03^a^	3.9 (1.4)	4.4 (1.1)	.001	3.4 (1.5)	4.1 (1.0)	.02^a^

^a^Not significant after false discovery rate correction.

^b^N/A: not applicable.

^c^5-point Likert scale (1=easy or not at all difficult to 5=very difficult).

^d^5-point Likert scale (1=very unlikely to 5=very likely).

**Figure 2:**
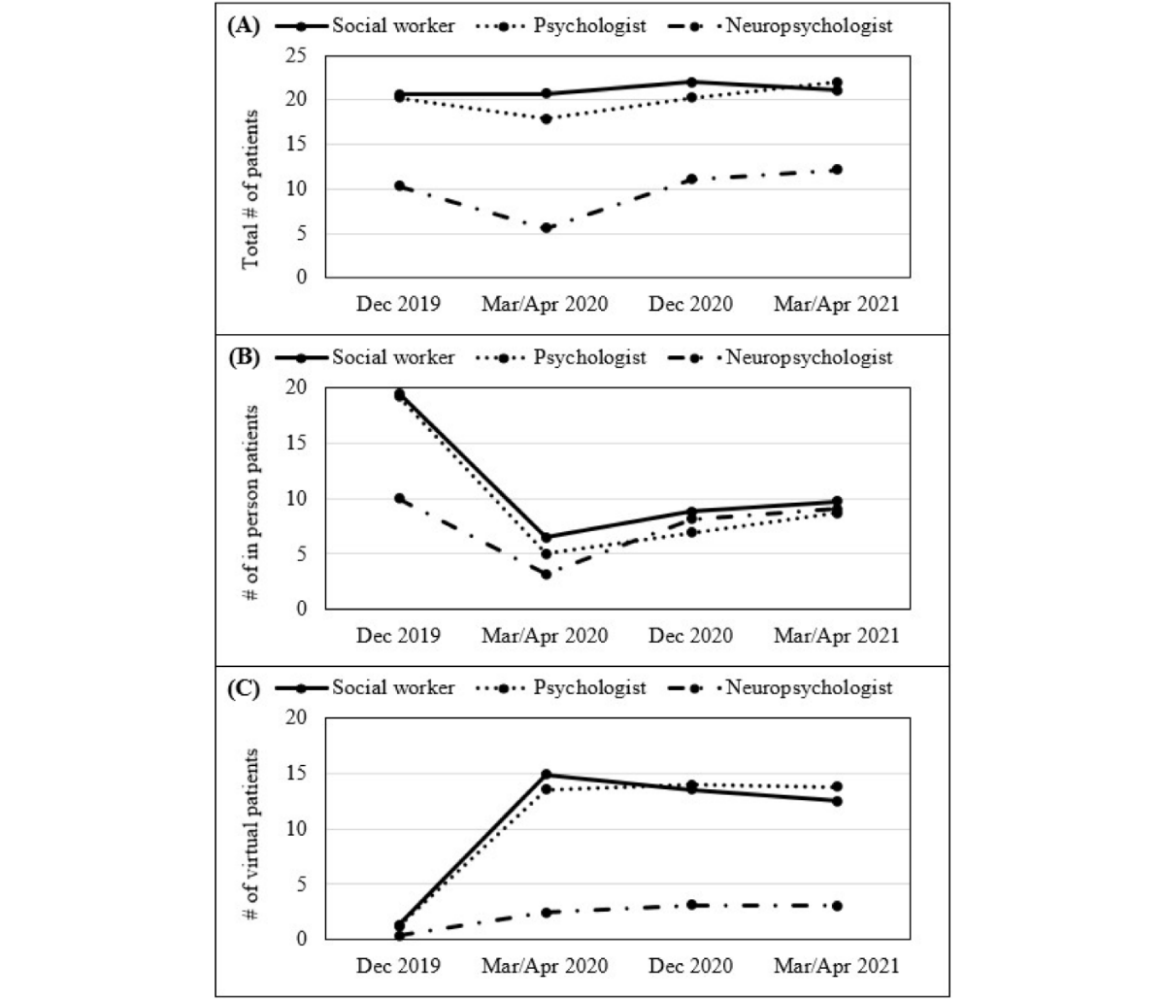
Changes in the number of patients seen weekly by provider type and location: (A) total patient population, (B) patients seen in person, (C) patients seen virtually.

The mixed ANOVA showed that the total number of patients seen for all providers decreased at the start of the COVID-19 pandemic (March 2020/April 2020) though increased by December 2020 (*F*_1.99,357.86_=8.09, *P*<.001; partial η^2^=0.04; Figure 2). Neuropsychologists saw fewer patients than psychologist/doctoral-level providers and social worker/master’s-level providers (*F*_2,180_=16.19, *P*<.001; partial η^2^=0.15), and the time x provider type interaction was not significant (*F*_3.98,357.86_=1.46, *P*=.22). For the number of patients seen in person, there was an initial decrease at the start of the COVID-19 pandemic, with an increase by December 2020, though the number of in-person patients did not return to prepandemic levels (*F*_2.26,372.02_=64.10, *P*<.001; partial η^2^=0.28). The time x provider type interaction was significant (*F*_4.51,372.02_=7.30, *P*<.001; partial η^2^=0.08) and showed that neuropsychologists saw fewer patients than other providers in December 2019, though this did not differ from other providers at subsequent time points. For the number of patients seen virtually, there was an initial increase at the start of the COVID-19 pandemic, which remained stable over time (*F*_2.37,376.04_=73.45, *P*<.001; partial η^2^=0.32). Neuropsychologists saw fewer patients overall than other providers (*F*_2,159_=28.93, *P*<.001; partial η^2^=0.27). The time x provider type interaction was significant (*F*_4.73,376.04_=11.50, *P*<.001; partial η^2^=0.13) and showed that, although there was no difference among providers in the number of patients seen virtually in December 2019, neuropsychologists saw fewer patients virtually than other providers across all other time points.

### Setting

Characteristics of the changes in practice by clinical practice setting are presented in Table 5. Only AMC providers had a significant decrease in the amount of time per week working remotely and in the percent of participants who reported implementing virtual instead of in-person visits as a practice adjustment. All settings had a decrease in the percent of appointments being rescheduled or postponed, though only AMCs and PP had a decrease in appointments being canceled. AMCs and PP reported an increase in implementation of “other” practice adjustments. “Other” practice adjustments for AMCs included safety precautions, hybrid models, offering telehealth as an option, and adjusting test batteries. Adjustments for PP included safety precautions, adding extra appointments, hybrid models, adjustments to test batteries, and reducing caseloads. Only AMCs had a significant increase in the percent of providers who were not implementing any changes to their current practice. Only AMCs had a significant decrease in difficulty with telemental health implementation. AMCs and PPs indicated an increased likelihood of continuing telemental health services in the future.

**Figure 3:**
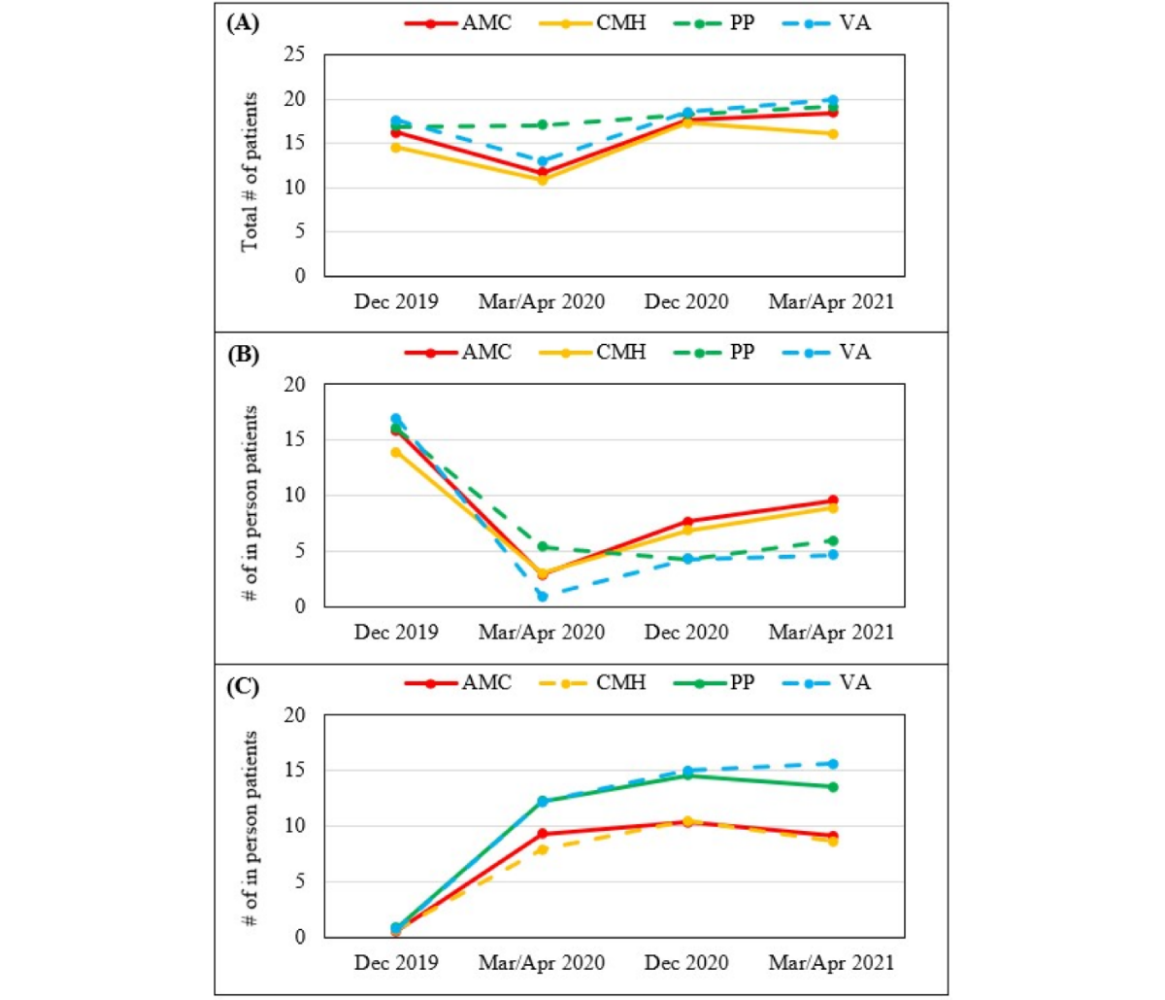
Changes in the number of patients seen weekly by setting and location: (A) total patient population, (B) patients seen in person, (C) patients seen virtually. AMC: academic medical center; CMH: community mental health; PP: private practice; VA: Veterans Affairs.

The mixed ANOVA showed that the total number of patients seen across settings decreased at the start of the COVID-19 pandemic (March 2020/April 2020), though it increased by December 2020 (*F*_2.08,298.79_=14.22, *P*<.001; partial η^2^ = 0.09; Figure 3). There were no differences across settings, and the time x setting interaction was not significant (*F*_6.23,298.79_=1.63, *P*=.14). For the number of patients seen in person, there was an initial decrease across settings at the start of the COVID-19 pandemic, with an increase by December 2020, though the number of in-person patients seen did not return to prepandemic levels (*F*_2.27,297.90_=64.39, *P*<.001; partial η^2^=0.33). The time x setting interaction was significant (*F*_6.82,297.90_=2.28, *P*=.03; partial η^2^=0.05), and the follow-up tests of simple main effects indicated that all settings had an increase in the number of patients seen in person after March 2020/April 2020 except for PPs. For the number of patients seen virtually, there was an initial increase at the start of the COVID-19 pandemic, which remained stable over time (*F*_2.40,304.15_=79.72, *P*<.001; partial η^2^=0.39). There were no differences across settings, and the time x setting interaction was not significant (*F*_7.19,304.15_=1.15, *P*=.33).

**Table 5:** Descriptive statistics of changes in clinical practice from Time 1 to Time 2 across settings.

Clinical practice		Academic medical center (n=52)				Community mental health (n=18)				Private practice (n=55)				Veterans Affairs (n=25)		
		Time 1	Time 2	*P* value	Time 1		Time 2	*P* value	Time 1		Time 2	*P* value	Time 1		Time 2	*P* value
**Practice adjustments, n (%)**																
	Telemental health or virtual appointments (vs in person)	48 (91.3)	31 (59.6)	<.001	14 (77.8)		14 (77.8)	≥.99	46 (83.6)		47 (85.5)	≥.99	24 (96.0)		23 (92.0)	≥.99
	Rescheduling or postponing appointments	29 (55.8)	3 (5.8)	<.001	10 (55.6)		0	.002	23 (41.8)		6 (10.9)	.001	10 (40.0)		0	.002
	Canceling appointments	16 (30.8)	3 (5.8)	.001	3 (16.7)		0	.25	11 (20.0)		1 (1.8)	.002	5 (20.0)		0	.06
	Restrictions on appointments	8 (15.4)	7 (13.5)	≥.99	2 (11.1)		2 (11.1)	≥.99	4 (7.3)		8 (14.5)	.34	0		6 (24.0)	.03^a^
	Other adjustment	1 (1.9)	10 (19.6)	.01	0		3 (16.7)	.25	1 (1.8)		12 (22.2)	.003	2 (8.0)		6 (25.0)	.22
	N/A^b^ (no change in practice)	1 (1.9)	11 (21.2)	.002	0		1 (5.6)	≥.99	1 (1.8)		2 (3.6)	≥.99	0		0	N/A
**Telemental health**																
	Reported not implementing telemental health currently, n (%)	5 (10.0)	6 (11.5)	≥.99	5 (27.8)		1 (5.6)	.13	1 (14.8)		4 (7.3)	.06	0		0	N/A
	Amount of week working remotely (%), mean (SD)	84.8 (27.6)	45.7 (39.1)	.002	77.9 (36.8)		67.2 (35.1)	.57	74.7 (36.6)		64.7 (38.0)	.10	83.8 (32.7)		75.0 (27.3)	.34
	Easy access to IT services, n (%)	49 (94.2)	51 (98.1)	.63	14 (77.8)		15 (83.3)	≥.99	19 (34.5)		15 (27.3)	.45	20 (80.0)		22 (88.0)	.63
	Difficulty with telemental health implementation^c^, mean (SD)	3.3 (1.1)	2.7 (1.0)	<.001	3.2 (1.2)		2.9 (1.1)	.21	2.5 (1.3)		2.4 (1.0)	.33	3.1 (1.2)		2.7 (1.2)	.11
	Likelihood of continuing to provide telemental health services^d^, mean (SD)	3.5 (1.2)	4.4 (0.8)	.001	3.3 (1.4)		3.8 (1. 3)	.19	3.5 (1.6)		4.3 (1.2)	.001	4.0 (1.4)		4.6 (0.6)	.07

^a^Not significant after false discovery rate correction.

^b^N/A: not applicable.

^c^5-point Likert scale (1=easy or not at all difficult to 5=very difficult).

^d^5-point Likert scale (1=very unlikely to 5=very likely).

## Discussion

### This Study

The COVID-19 health crisis was far-reaching, and adjustments in the way clinicians and patients interfaced with health care were necessary. Mental health services were highlighted as crucial throughout the pandemic given the broad day-to-day life changes required to reduce transmission of the virus. During our initial survey [23], we identified significant mental health practice changes and highlighted the implementation of telehealth, which was utilized sporadically prior to the pandemic. With this study, we hoped to create a longitudinal understanding of mental health practices as the pandemic became an everyday part of life. Others have also discovered that the hybrid model of mixed telehealth and in-person services is well accepted by mental health practitioners and their patients [28]. Given the initial findings, we predicted that there would be continued acceptance of adopting telemental health services, fewer canceled appointments, easier access to telehealth technology, increased access to mental health services, and an increase in in-person visits as pandemic restrictions were lifted.

### Principal Findings

A year into the COVID-19 pandemic, mental health practitioners reported a significant decrease in the amount of time working remotely, and fewer providers reported offering virtual instead of in-person visits. However, despite some decrease in reliance on telemental health services, the overall number of providers using telemental health services continued to increase, and nearly all providers reported using telemental health in some capacity. Providers continued to work an average of 3 days per week remotely, which is consistent with prior work suggesting that mental health services are moving to a hybrid model of practice [8,9,24-26]. Hybrid models can offer greater flexibility for both patients and providers, though they can pose challenges regarding security of health information, reduced informal social interaction, and access to reliable technology. Although participants in our study reported decreased difficulty with telemental health implementation, this decrease was most notable for psychologists and AMC providers, who may have additional resources that are not readily available to providers in a PP setting. Importantly, providers overall endorsed an increased likelihood of continuing to provide telemental health services in the future, which highlights that continued refinement of infrastructure in the workplace and home to support these hybrid models is crucial.

The change in patients seen throughout the first year of the COVID-19 pandemic was consistent with predictions. There was an initial decline in the total number of patients and number of in-person patients seen from shortly prior to the start of the pandemic (December 2019) to shortly after most lockdown protocols were implemented (March 2020/April 2020). This coincided with an increase in the number of patients seen virtually. Although the total number of patients seen weekly increased back to prepandemic levels, this was primarily driven by the increase in the number of patients seen virtually, as the number of patients seen in person did not yet return to prepandemic levels by March 2021/April 2021. This increase was consistent with providers’ reporting canceling and rescheduling fewer in-person appointments compared with the start of the pandemic, which was likely related to relaxation of initial stay-at-home orders and implementation of clinic procedures to reduce the risk of transmission. Providers continued to implement restrictions on appointments and other methods of infection control, such as policies regarding recent travel, sanitization practices, and symptom screening, which may have resulted in greater comfort with increasing in-person visits. It is important to note that the number of patients seen virtually remained stable from March 2020/April 2020 to March 2021/April 2021, which resulted in a general trend of more patients being seen overall as the number of in-person patients increased. This finding could be related to increased provider availability due to improved efficiency and comfort with telehealth services as the pandemic progressed [18]. It is also possible that this trend may be due to greater prevalence of mood symptoms resulting in higher demand for mental health services throughout the pandemic [29,30]. It will be important to continue monitoring whether this increase in the total number of patients continues to increase, as increased burden on the health care workers due to the COVID-19 pandemic has contributed to significant burnout and may reduce quality of care provided [31,32].

There were also several findings that were unique to certain settings and provider types. Neuropsychologists saw fewer total patients and virtual patients when compared with social workers and psychologists, though the number of patients seen in person after the start of the pandemic in March 2020/April 2020 did not differ. This was a somewhat expected finding given the time each provider might devote to a single patient (eg, 2-8 hours of neuropsychological assessment for 1 patient versus 1-hour sessions with numerous cases per day), though this highlights unique challenges that neuropsychologists may have had regarding utilizing telemental health in their practice. Patient interviews and feedback sessions are readily adaptable to a virtual format, though procedures for conducting testing in a virtual format are still being developed and are not widely implemented [33]. Among settings, providers who worked at AMCs initially reported the highest amount of time worked remotely (85% per week), though this decreased to below the overall sample mean by March 2021/April 2021. It is possible that certain rules and regulations within settings may contribute to greater pressure to return to a prepandemic baseline. It is also possible that greater resources within a larger system allowed providers to continue the prior standard of care, as AMC providers also reported greater likelihood of continuing to provide telemental health services despite a significant decrease in offering virtual instead of in-person appointments. Additionally, providers in PP did not show the expected increase in patients seen in person between March 2020/April 2020 and March 2021/April 2021. This could be due to less of an initial decline from December 2019 to March 2020/April 2020, as the salary for PP providers is typically more dependent on the number of patients seen compared with other settings with a more fixed salary schedule. Last, although providers in most settings reported a reduction in canceling or rescheduling appointments, all providers in Veterans Affairs hospitals and community mental health settings reported that no appointments were rescheduled nor canceled by March 2021/April 2021. These settings also both had no change in continuing to implement virtual instead of in-person visits as a practice adjustment, which could have contributed to greater flexibility in the type of appointments offered if a patient reported a COVID-19 exposure or onset of symptoms.

### Limitations

Longitudinal survey data have a number of common limitations. Given the longitudinal nature of the data in this study, 37.5% of the individuals who provided their email addresses during the initial survey responded to the current follow-up survey. Additionally, some individuals changed practice setting, which we accounted for in our analyses but also impacts the quality of the longitudinal data. An important limitation of survey data is that the data are based on self-report, and we asked for retrospective estimates of the number of patients seen in December 2019 and December 2020. As such, the increase in the total number of patients with stable numbers of telehealth services could also suggest that some of the initial estimates were low or a reflection of changes in the overall practices within mental health specialties (eg, current billing rates compared with current inflation rates). Although our survey had similar characteristics to other surveys and was representative of the original sampling distribution, the demographics of participants who responded to the survey may not be representative of all mental health providers. Participants in our study were predominantly female (87%) and White (90%), and nearly one-half of participants were from the southern United States. As such, these results may not reflect practice changes in other regions of the United States, such as the Northeast, which had varying patterns of COVID-19 mortality and vaccination compared with the South [34].

### Conclusions

Hybrid in-person and telemental health services appear to be here to stay. These results show that the rapid transition to telemental health at the start of the COVID-19 pandemic in spring 2020 was sustained over the next year, despite an overall increase in the number of patients seen in person. Although more providers reported returning to working on-site, over 50% of providers continued to use a hybrid model, and many providers reported they would be more likely to continue telemental health beyond spring 2021. Our findings suggest that it will be important to continue to provide support and resources to successfully implement a hybrid telehealth model. This hybridization of mental health is potentially allowing for an overall increase in access to services, which is particularly important given the mental health impacts of the pandemic. Additionally, easy access to IT infrastructure and education may facilitate adoption of hybrid practice models across settings.

## Appendix

Multimedia Appendix 1 Survey.

## Abbreviations

AMC: academic medical center
PP: private practice
T1: Time point 1
T2: Time point 2

## Data Availability

The data sets generated during and/or analyzed during this study are available from the corresponding author on reasonable request.
