# Supplementary material for: Changes in the Clinical Practice of Mental Health Service Providers Throughout the COVID-19 Pandemic: Longitudinal Questionnaire Study
Source: JMIR Form Res. 2024 Apr 29;8:e50303. doi: 10.2196/50303 (PMC11060325; doi:10.2196/50303)
Supplement: Multimedia Appendix 1 [file formative_v8i1e50303_app1.docx]

Appendix 1. Survey

**Follow-up National Survey: Mental Health Practitioners’ Response to COVID-19**

*Thank you for your interest and participation in this survey, as well as your previous participation in the survey you completed in early 2020. Similar to the prior survey, in this survey you will be asked information pertaining to personal characteristics and your current practical responses to COVID-19, as well as your current perceptions of your institution’s response, your current emotional response, and your current perceptions about COVID-19. Questions that ask if there have been any changes since April 2020 are intended to distinguish whether there have been any changes since the approximate time that you completed the initial survey.*

*Your involvement in this project is completely voluntary, you can discontinue participating at any time while completing the survey, and you do not have to respond to questions you prefer not to answer. All information provided will be reported on as aggregated, de-identified data. Your email address will be stored separately from your responses and linked using a unique identifier. No one other than the study team will have access to this information. If you choose to provide your email, it will only be used as a way to contact you for a follow-up survey, which you do not have to complete.*

**The following questions ask about your personal information and information about patients seen in your practice.**

1. Email address (from which you received this survey request): _____________________
2. What is your age?
3. What is your highest level of education?
   1. High school or equivalent (e.g., GED)
   2. Associate’s degree
   3. Bachelor’s degree
   4. Master’s degree
   5. Doctoral degree

3a. Has your education status changed since April 2020?

- - - - 1. Yes
        2. No

3aa. If Yes, why was there a change?

1. What is your approximate gross household income?
2. Less than $20,000
3. $20,000 – $39,999
4. $40,000 – $59,999
5. $60,000 – $79,999
6. $80,000 – $99,999
7. $100,000 – $119,999
8. $120,000 – $139,999
9. $140,000 – $159,999
10. $160,000 – $179,999
11. $180,000 – $199,999
12. $200,000 or more

4a. Has your gross household income changed since April 2020?

1. Yes
2. No
3. What is your relationship status?
   1. Single
   2. In a relationship, cohabitating
   3. In a relationship, not cohabitating
   4. Married/civil union
   5. Divorced
   6. Separated
   7. Widowed

5a. Has your relationship status changed since April 2020?

1. Yes
2. No
3. In which U.S. state/district/territory are you employed?

6a. Has your state of employment changed since April 2020?

1. Yes
2. No

6b. How has your employment status changed since April 2020:

1. In what type of employment are you currently engaged?
   1. Full time
   2. Part time
   3. N/A, not currently employed
   4. Other, please specify _________

7a. Has your type of employment changed since April 2020?

1. Yes
2. No
3. Current provider level (please select one):
   1. Graduate-level practicum student
   2. Pre-doctoral intern
   3. Postdoctoral fellow
   4. Unlicensed practitioner
   5. Licensed practitioner
   6. Licensed practitioner and board-certified in specialty area

8a. Has your provider status changed since April 2020?

- - - - 1. Yes
        2. No

8b. Were you a trainee (i.e., practicum student, pre-doctoral intern, postdoctoral fellow) in April 2020?

1. Yes
2. No
3. What type of provider are you? Please select one option that fits best.
   1. Bachelor’s level therapist/counselor
   2. Social worker or master’s-level therapist/counselor
   3. Psychologist or doctoral-level therapist/counselor
   4. Neuropsychologist
   5. Marriage and Family/Couple Therapist (MFTs)
   6. Psychiatrist
   7. Other physician, please specify______
   8. Psychiatric nurse practitioner or psychiatric physician assistant
   9. Support staff
   10. Other, please specify_______
4. What is your current practice setting? Select all that apply.
   1. Academic Medical Center
   2. Community Mental Health Setting
   3. Department/Graduate Training Clinic
   4. General Hospital
   5. Law Firm
   6. Prison
   7. Private Practice
   8. Psychiatric Hospital or Facility
   9. Rehabilitation Hospital or Setting
   10. School
   11. University Counseling Center
   12. Veterans Hospital or Military Hospital/Clinic
   13. Other, please specify______
5. How many patients did you see **per week** ***in December 2020***?
   1. In person
   2. Remote/telehealth
6. How many patients are you ***currently*** seeing **per week** (i.e., the week you are completing this survey?
   1. In person
   2. Remote/telehealth
7. Please include any additional information that you feel would be beneficial and/or clarify your responses above, ***related to your personal information or patients seen in your practice***.

**The following questions will ask about your response to COVID-19 as a provider.**

1. At the current time, how have you adjusted your practice with patients following the COVID-19 pandemic, if at all? Please select all that apply.
   1. N/A, no change in practice
   2. Cancelling patient appointments
   3. Rescheduling/postponing patient appointments
   4. Telehealth/virtual appointments instead of in-person appointments
   5. Restricting types of patients who are scheduled (e.g., based on age, medical comorbidity, recent travel)
   6. Other adjustment to practice, please specify: _____________
2. How far out are you rescheduling/postponing patients on average? Please select the best option.
3. Two-three weeks
4. One month
5. Two months
6. Three months
7. Indefinitely
8. What type of telehealth are you using? Select all that apply.
9. Audio
10. Audio and video
11. Secure email
12. Other, please specify ______
13. On what bases are you restricting patient visits? Please select all that apply)?
14. Age (e.g., if a patient is above a certain age)
15. Medical comorbidities
16. Recent travel
17. Physical symptoms (e.g., if a patient has a cough, fever, shortness of breath)
18. Crisis care (e.g., patients who are actively suicidal)
19. Other, please specify: _______
20. Select the range of ages for patients you ARE continuing to see in your practice.
21. Please select the medical comorbidities you are considering in restricting patient visits currently. Select all that apply.
    1. Cardiovascular/cerebrovascular disorders (e.g., diabetes, coronary artery disease)
    2. Lung disease (e.g., COPD)
    3. Immunocompromised/immunosuppressed (e.g., HIV, multiple sclerosis)
    4. Renal disease (e.g., chronic kidney disease)
    5. Other medical comorbidity, please specify: __________
22. Please select the recent travel criteria on which you are currently restricting patient visits. Select all that apply.
23. Out-of-state domestic travel
24. International travel
25. For neuropsychologists only: Please select the types of evaluation services you are currently providing. Select all that apply.
26. Not currently providing any services secondary to COVID-19-related concerns
27. Inpatient/consults
28. In-person, interview only
29. In-person, interview and testing
30. Telehealth/virtual appointment, interview only
31. Telehealth/virtual appointment, interview and testing
32. For neuropsychologists only: If “Telehealth/virtual appointment, interview and testing” is selected, Please specify the measures you are administering remotely/via telehealth.
33. Are you currently offering services via telehealth/virtual appointments?
    1. Yes
    2. No

23a. Had you previously been offering services via telehealth/virtual appointments?

- - - - 1. Yes
        2. No

23aa. Why did you discontinue?

1. Returned to only in-person appointments
2. Provider barriers in continuing telehealth/virtual appointments (e.g., technical difficulties, workflow issues)
3. Patient barriers in continuing telehealth/virtual appointments (e.g., insufficient Internet or device access, lack of patient interest in telehealth/virtual appointments)
4. Patient dissatisfaction
5. Provider dissatisfaction
6. Billing issues/Insurance Reimbursement (please specify): ________
7. Administrative issues (please specify): ________
8. Other (please specify): ________
9. How difficult has it been to implement telehealth/virtual appointments?
   1. Easy/not at all difficult
   2. Somewhat easy
   3. Neutral (not easy or difficult)
   4. Somewhat difficult
   5. Very difficult
   6. N/A have not implemented telehealth/virtual appointments during the COVID-19 pandemic
10. Do you work in a setting with easy access to information technology (IT) staff/services?
    1. Yes
    2. No
11. If it remains available, how likely are you to continue providing services via telehealth/virtual appointments?
    1. Very unlikely
    2. Somewhat unlikely
    3. Neutral (not likely or unlikely)
    4. Somewhat likely
    5. Very likely
12. What additional therapeutic services, if any, are you providing to existing/new patients ***related specifically to COVID-19 concerns***? Please select all that apply.
    1. N/A, no additional therapeutic services
    2. Individual therapy to support existing/new patients
    3. Individual therapy to support to medical providers specifically
    4. Family therapy to support existing/new patients
    5. Family therapy to support medical providers specifically
    6. Group therapy to support existing/new patients
    7. Group therapy to support medical providers specifically
    8. Providing resources (e.g., pamphlets) to existing/new patients
    9. Community outreach (e.g., consulting with community agencies)
    10. Non-clinical support group (e.g., social media page, virtual lunch hours, peer support pairing)
    11. Crisis care
13. Are you providing crisis care related to COVID-19 concerns (select all that apply):
    1. In person
    2. Remotely/via telehealth
14. What percentage of your week are you **currently** working at home/remotely?
15. Are you currently engaging in supervision/consultation/peer support groups with colleagues related to COVID-19 concerns?
    1. Yes
    2. No
16. Please include any additional information which you would feel would be beneficial and/or clarify your responses above, ***related to your response to COVID-19 as a provider***.

**The following questions will ask about your perceptions of your institution/employer/ practice’s response to COVID-19.**

1. My institution/employer/practice has placed the following restrictions on coming into work. Please select all that apply.

|  |  | Not restricted or N/A | Previous Restriction | Current Restriction |
| --- | --- | --- | --- | --- |
| a. | N/A no restrictions |  |  |  |
| b. | Travel-related restrictions |  |  |  |
| c. | Symptom-related restrictions (e.g., if an employee has a cough, fever, shortness of breath) |  |  |  |
| d. | Age-related restrictions (e.g., if an employee is over a certain age) |  |  |  |
| e. | Restrictions based on medical comorbidities (e.g., chronic illnesses, immunocompromise) |  |  |  |
| f. | No one is allowed to come into work |  |  |  |
| g. | Other, please specify: ____________ |  |  |  |

1. Please specify travel-related restrictions on coming into work placed by your institution/employer/practice. Select all that apply.
   1. Out-of-state domestic travel
   2. International travel
2. Please select the range of ages for employees who **ARE NOT RESTRICTED** from coming into work
3. To what extent have you followed your work-place travel-related restrictions?
   1. I have followed travel-based restrictions completely.
   2. I traveled out of state/country during the period of travel restriction, but received permission from my immediate supervisor or superior.
   3. I traveled out of the state/country during the period of travel restriction without permission from my employer.
   4. N/A, I had no plan to travel regardless of restrictions.
4. Please respond regarding the degree to which you agree with each statement.

|  |  | Strongly disagree | Somewhat disagree | Neutral | Somewhat agree | Strongly agree |
| --- | --- | --- | --- | --- | --- | --- |
| a. | Social distancing (e.g., 6 feet between people) has been practiced reliably in my work environment. |  |  |  |  |  |
| b. | My institution/employer/ practice has been adequately prepared to address concerns/changes arising from COVID-19. |  |  |  |  |  |
| c. | My institution/employer/ practice has been responding appropriately to COVID-19. |  |  |  |  |  |
| d. | My institution/employer/ practice has been providing a safe working environment during COVID-19. |  |  |  |  |  |
| e. | I am satisfied with the amount of information my institution/employer/ practice has provided me about COVID-19 and associated institutional changes. |  |  |  |  |  |
| f. | Communications received from various people in my institution/practice regarding responses to COVID-19 have been consistent. |  |  |  |  |  |
| g. | My institution/employer/ practice has provided appropriate precautionary materials (e.g., hand sanitizer, gloves, masks) in my work environment. |  |  |  |  |  |
| h. | My institution/employer/ practice has provided appropriate logistical/living provisions (e.g., pay, leave to care for a dependent). |  |  |  |  |  |
| i. | My institution/employer/ practice has provided adequate information/ training about providing telehealth. |  |  |  |  |  |
| j. | My institution/employer/ practice has provided adequate information/ training about providing crisis care remotely. |  |  |  |  |  |

1. How effective do you think the institutional measures employed by your institution/employer/practice have been overall since the pandemic began?
   1. Very ineffective
   2. Somewhat ineffective
   3. Neutral (not effective or ineffective)
   4. Somewhat effective
   5. Very effective

37a. Since April 2020, do you feel as if your institution/employer/practice’s institutional measures related to the pandemic have:

- - - - 1. Improved greatly
        2. Improved somewhat
        3. Stayed the same
        4. Worsened somewhat
        5. Worsened greatly

1. For trainees only (e.g., participants who select that they are currently a graduate-level practicum student, intern, or postdoctoral fellow): Please respond regarding the degree to which you agree with each statement.

|  |  | Strongly disagree | Somewhat disagree | Neutral | Somewhat agree | Strongly agree |
| --- | --- | --- | --- | --- | --- | --- |
| a. | Information and planning to respond to COVID-19 have been adequately disseminated to me by my supervisor(s). |  |  |  |  |  |
| b. | My supervisor(s) have been responsive to my questions and concerns regarding operational/training changes related to COVID-19. |  |  |  |  |  |

38a. Since April, 2020, do you feel as if your supervisor(s)’ communication related to the pandemic has:

- - - - 1. Improved greatly
        2. Improved somewhat
        3. Stayed the same
        4. Worsened somewhat
        5. Worsened greatly
        6. Not applicable (no longer same supervisor, different position, etc.)

1. Please include any additional information that you would feel would be beneficial and/or clarify your responses above, ***related to your institution/employer/practice’s response to COVID-19***.

**The following questions will ask about your emotional response to COVID-19.**

1. Please describe your personal experience(s) related to COVID-19. Please select all that apply.
   1. N/A, I have not had a personal experience with COVID-19
   2. I had symptoms but was not tested
   3. I had symptoms and had a negative test (i.e., no virus)
   4. I had symptoms and had a positive test (i.e., virus present)
   5. I have been hospitalized related to COVID-19.
   6. Someone close to me had a positive test (i.e., virus present)
   7. Someone close to me was hospitalized related to COVID-19.
   8. Someone close to me has died related to COVID-19.
2. Please rate your **current** level of general anxiety/distress ***associated with the COVID-19 pandemic***.

41a. Do you feel as if your anxiety/distress associated with the COVID-19 pandemic has changed since April 2020?

1. Decreased greatly
2. Decreased somewhat
3. No change
4. Increased somewhat
5. Increased greatly
6. Please rate your **current** level of anxiety/distress ***that you or someone you know will contract COVID-19***.

42a. Do you feel as if your anxiety/distress that you or someone you know will contract COVID-19 has changed since April 2020?

1. Decreased greatly
2. Decreased somewhat
3. Stayed the same
4. Increased somewhat
5. Increased greatly
6. Please rate your **current** level of anxiety/distress ***associated with societal impacts of COVID-19*** (e.g., related to mental health, capacity of healthcare system to accommodate affected individuals, the economy). *(Sliding scale 0-10, higher number = more anxiety/distress)*

43a. Do you feel as if your anxiety/distress associated with societal impacts of COVID- 19 has changed since April 2020?

1. Decreased greatly
2. Decreased somewhat
3. Stayed the same
4. Increased somewhat
5. Increased greatly
6. Are you a primary caregiver (e.g., of a child, parent, parent-in-law, individual with a disability)?
   1. Yes
   2. No

44a. Did your caregiver status change since April 2020?

1. Yes
2. No
3. Please rate your level of anxiety/distress related to childcare/eldercare/ adult supervision related to COVID-19.

45a. In comparison to April 2020, do you feel as if your current level of anxiety/distress related to childcare/eldercare/adult supervision has:

1. Decreased greatly
2. Decreased somewhat
3. Stayed the same
4. Increased somewhat
5. Increased greatly

1. To what extent do you think that your anxiety/distress related to COVID-19 currently affects your ability to provide services to patients?
   1. N/A, I am not currently working/providing patient services secondary to COVID-19-related concerns
   2. No impact
   3. Very small impact
   4. Somewhat small impact
   5. Somewhat large impact
   6. Very large impact
2. To what extent are you concerned that changes related to COVID-19 has affected or will affect your training experiences/expectations?
   1. Very Unconcerned
   2. Somewhat Unconcerned
   3. Neutral (neither concerned or unconcerned)
   4. Somewhat concerned
   5. Very concerned
3. For trainees or recent trainees (<1 year ago) only: To what extent have the changes related to COVID-19 **actually** affected your training experiences/expectations?
   1. No impact
   2. Very small impact
   3. Somewhat small impact
   4. Somewhat large impact
   5. Very large impact
   6. N/A, not a current or recent (<1 year ago) trainee
4. Since the beginning of the pandemic, what coping strategies have you employed to manage/alleviate anxiety/distress associated with COVID-19? Please select all that apply.
   1. No coping strategy
   2. Individual therapy/counseling
   3. Group therapy/counseling
   4. Supervision
   5. Peer consultation
   6. Distraction/engaging in an enjoyable activity
   7. Spending time with loved ones (in person or virtual)
   8. Exercise
   9. Therapeutic strategies
   10. Relaxation/relaxation apps
   11. Avoiding the feelings
   12. Rationalizing
   13. Education/information
   14. Media/social media restrictions
   15. Religion/faith/spirituality
   16. Alcohol
   17. Tobacco
   18. Other substance use
       1. Please specify all other substance(s) used: _____
   19. Other strategy, please specify __________
5. For each coping strategy selected above, how effective has the selected coping strategy been in managing/alleviating your anxiety/distress related to COVID-19? (choices offered for each strategy selected in the previous question)
   1. Very ineffective
   2. Somewhat ineffective
   3. Neutral (not effective or ineffective)
   4. Somewhat effective
   5. Very effective
6. How often, on average, have you used alcohol during the pandemic?
   1. 0 = Never
   2. 1 = Monthly or less
   3. 2 = 2-4 times a month
   4. 3 = 2-3 times a week
   5. 4 = 4 or more times a week
7. During a typical time when you have used alcohol during the pandemic, approximately many standard drinks did you have?
   1. 0 = 1 or 2
   2. 1 = 3 or 4
   3. 2 = 5 or 6
   4. 3 = 7 to 9
   5. 4 = 10 or more
8. How have you used alcohol during the pandemic? Select all that apply.
   1. Socially (e.g., when others were drinking alcohol as well) either in person or virtually (e.g. Zoom or FaceTime).
   2. In the company of others who were not drinking alcohol in person or virtually
   3. Alone
9. How have you ***typically*** used alcohol during the pandemic? Note that socially can be in person or virtually.
   1. Only socially (e.g., others were always present when you used alcohol)
   2. Mostly socially, with some drinking alcohol alone
   3. Equally socially and alone
   4. Mostly alone, with some drinking alcohol socially
   5. Only alone (e.g., others were never present when you used alcohol)
10. In comparison to April 2020, do you feel as if your alcohol use has:
    1. Decreased significantly
    2. Decreased somewhat
    3. Stayed the same
    4. Increased somewhat
    5. Increased significantly

55a. Prior to April 2020, how did you ***typically*** use alcohol during the prior year? Note that socially can be in person or virtually.

- - - - 1. Only socially (e.g., others were always present when you used alcohol)
        2. Mostly socially, with some drinking alcohol alone
        3. Equally socially and alone
        4. Mostly alone, with some drinking alcohol socially
        5. Only alone (e.g., others were never present when you used alcohol)

**The following questions will ask about your perceptions about COVID-19.**

1. When do you think the COVID-19 Pandemic will be resolved (i.e., return to pre-COVID-19 day-to-day functions) in the United States?
   1. Sooner than summer 2021
   2. By summer 2021
   3. By fall 2021
   4. After fall 2021
   5. Another time, please specify: ________
2. What is the main source of your health information about COVID-19? Please select one.
   1. Internet
   2. Television
   3. Radio
   4. Social media
   5. Print Media
   6. Friends/family members
   7. Employer
   8. Other sources, please specify: ____________
3. How satisfied are you with the amount of health information available about COVID-19?
   1. Very unsatisfied
   2. Somewhat unsatisfied
   3. Neutral (not satisfied or unsatisfied)
   4. Somewhat satisfied
   5. Very satisfied
4. Health information provided to me about COVID-19 affected me in the following ways. Please select all that apply.
   1. N/A, health information provided to me about COVID-19 has not affected me.
   2. Led me to change my behavior in an adaptive way
   3. Led me to change my behavior in a maladaptive way
   4. Led me to worry less
   5. Led me to worry more
   6. Led me to be a better provider
   7. Led me to be a worse provider
   8. Other, please specify: ___________
5. Related to the COVID-19 vaccine, I:
   1. Received the first dose of the vaccine
   2. Received both the first and second doses of the vaccine
   3. Am signed up to get my first dose of the vaccine
   4. Plan to get vaccinated when it becomes available at me
   5. Do not plan to get vaccinated
   6. I am unsure whether I will get vaccinated.
6. For what reason(s) are you unsure whether you will get vaccinated??
7. Where did you get vaccinated?
   1. Through my workplace
   2. In the community (e.g., pharmacy, hospital, county or state sponsored vaccination site)
8. When did you get the first dose of the vaccine?
   1. December 2020
   2. January 2021
   3. February 2021
   4. March 2021
   5. April 2021
9. Please rate the extent to which you agree with the following statements:

|  |  | Strongly disagree | Somewhat disagree | Neutral | Somewhat agree | Strongly agree |
| --- | --- | --- | --- | --- | --- | --- |
| a. | Getting vaccinated will reduce the likelihood that someone will contract COVID-19. |  |  |  |  |  |
| b. | Wearing a mask reduces the likelihood that the mask wearer will contract COVID-19. |  |  |  |  |  |
| c. | Wearing a mask reduces the likelihood that others will contract COVID-19. |  |  |  |  |  |
| d. | It is important to wear a mask indoors when around other people not in your household. |  |  |  |  |  |
| e. | It is important to wear a mask outdoors when less than six feet apart from other people not in your household. |  |  |  |  |  |
| f. | It is important to wear a mask outdoors when you are more than 6 feet away from other people not in your household. |  |  |  |  |  |
